# Colonizable probiotic *Lactobacillus paracasei* R3 enhances ICI therapy *via* modulating PBMCs differentiation

**DOI:** 10.3389/fmicb.2025.1547964

**Published:** 2025-06-04

**Authors:** Wencan Song, Jizhen Liu, Jingfang Yang, Tao Chen, Jiayi Zhu, Xia Liu

**Affiliations:** ^1^Department of Oncology, The People’s Hospital of Chizhou, Chizhou, Anhui, China; ^2^Laboratory Animal Center, Guangdong Medical University, Dongguan, Guangdong, China; ^3^Center of Human Microecology Engineering and Technology of Guangdong, Guangzhou, Guangdong, China; ^4^Hubei Key Laboratory of Pollutant Analysis and Reuse Technology, College of Chemistry and Chemical Engineering, Hubei Normal University, Huangshi, China

**Keywords:** *Lactobacillus paracasei* R3, ICI therapy, colonization, metabolite, tumor inhibition

## Abstract

*Lactobacillus paracasei* R3 (*L.p* R3) has been reported to be effective to improve anti-tumor therapy for anti-tumor treatment in immune checkpoint inhibitor (ICI) therapy for colorectal cancer. However, the mechanisms behind this remain unclear. The present study shows that *L.p* R3 significantly enhanced anti-tumor efficacy ICI therapies in various tumor. Our results also showed the rapid adherence of *L.p* R3 to intestinal epithelial cell membrane, and promoted intestinal epithelial cell expression of mucin mRNA, led to the long maintenance of *L.p* R3 in colon and cecum in mice. *L.p* R3 significantly elevated the levels of macrophages, CD4^+^T cells and CD8^+^T cells in PBMCs while simultaneously decreasing the level of programmed cell death protein 1 (PD-1) on the surface of CD4^+^T cells and CD8^+^T cells. In addition, indole-3-carboxaldehyde, a metabolite of *L.p* R3 serves as an aryl hydrocarbon receptor (AHR) ligand regulating immune responses, was significantly upregulated in the serum of mice orally treated with *L.p* R3. In summary, our findings provide novel insights into the immune regulation of probiotics in anti-tumor responses and present a potential avenue for *L.p* R3 in promoting ICI efficacy.

## 1 Introduction

Immune checkpoint inhibitor (ICI) plays a vitally important role in cancer treatment, which has shown to be effective to combat different types of cancer including small cell lung cancer (Sundar et al., 2015), melanoma (Hamid et al., 2013; Larkin et al., 2015), carcinoma of urinary bladder (Reuters, 2017), and liver cancer (El-Khoueiry et al., 2017) by activating anti-tumor immunity. Although the inhibition to inhibitory T cell receptors such as programmed cell death protein 1 (PD-1) and its ligand PD-L1 can induce anti-tumor T cell responses, most of tumor patients shown response to single-drug therapy by ICI at a low rate (20%–40%) (Rodriguez-Pascual et al., 2019). Moreover, ICI therapy results in some side effects such as low response rate of ICIs, secondary drug resistance, and immune-related adverse reactions (Sivan et al., 2015). Given the low efficacy and side effects of ICI therapy, there remains a need for an improvement of immune treatments for tumor patients in clinical medicine.

Gut microbiota (GM) was considered important in the regulation of immune response (Patrick et al., 2018; Viaud et al., 2013). The discovery of the effects of GM on immune regulation was considered to be one of the five greatest discoveries in 2018 (Matson et al., 2018; Postow et al., 2018). Previous study has shown that probiotics can enhance gut barrier function and trigger immune response by activating dendritic cells (DCs), macrophages, CXCR4, MHC-1, and Th7/T-reg (Lin et al., 2023). Meanwhile, probiotics have shown to enhance immune-mediated anti-tumor activity, thereby improving selective killing of tumor cells. Many studies have demonstrated the importance of Lactobacillus in immune regulation (Bender et al., 2023; Zhang et al., 2023). For example, *L. paracasei* sh2020 has shown a synergetic anti-tumor effect with anti-PD1 by enhancing the anti-tumor function and intestinal barrier function, which induces the up-regulation of CXCL10 expression in tumor and subsequently increases the recruitment of CD8^+^T cells (Zhang et al., 2022). L. CASEI 431^®^ could promote the differentiation of CD4^+^T cells into Th17 cells in mouse Peyer patches, thereby activating immune cells and enhancing immunity (Ren et al., 2020).

Our previous works have been demonstrated that *Lactobacillus paracasei* R3 (*L.p* R3), a bacteria isolated from the feces of healthy baby, is effective in immune regulation and GM regulation. *L.p* R3 strain has been reported to ameliorate colitis of mice, decrease inflammatory cell infiltration, inhibit Th17 cell activity, and simultaneously enhancing Treg cells function (Huang et al., 2021). In addition, *L.p* R3 strain was also found to be effective in MC38 colon cancer prevention and treatment while significantly enhancing ICI efficacy in MC38 colon cancer (Chen et al., 2023). Although ICI therapy gave a higher efficacy in the presence of *L.p* R3, the precise mechanisms behind this remain unclear.

The aim of this study is to gain a better understanding of how *L.p* R3 promotes the efficacy of ICI therapy to suppress tumor development. Our results suggested that the synergetic use of *L.p* R3 and PD-1 inhibited the development of various tumors. *L.p* R3 was mainly colonized in cecum and colon of mice after oral treatment followed by adhesion on the membrane of intestinal epithelial cells, which further promoted enterocyte secretion of adhesion molecules. More importantly, *L.p* R3 altered the differentiation of Peripheral Blood Mononuclear Cells (PBMCs) and promoted immune cytokines secretion by PBMCs. Finally, *L.p* R3 significantly promoted the upregulation of 3-indole formaldehyde, a tryptophan metabolite in mice serum.

## 2 Results

### 2.1 *L.p* R3 strain characterization

**FIGURE 1:**
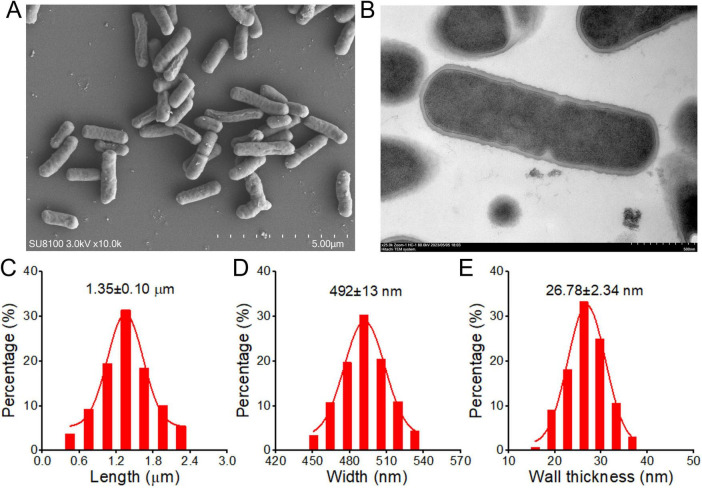
The Scanning **(A)** and Transmission **(B)** electron microscope images of *Lactobacillus paracasei* R3 (*L.p* R3). The bacterial length **(C)**, width **(D)** and bacterial wall thickness **(E)** of *L.p* R3.

To characterized the *L.p* R3, electron microscopy work was undertaken to observe *L.p* R3 micromorphology. Scanning electron micrographs of *L.p* R3 revealed the rod-shape of *L.p* R3 strain with irregular surfaces (Figure 1A), showing an average length of 1.35 μm (Figure 1C) and width of 492 nm (Figure 1D). Transmission electron micrographs of *L.p* R3 revealed a thinnish bacterial wall surrounding *L.p* R3 (*ca*.26.8 nm in thickness) (Figures 1B, D).

### 2.2 *L.p* R3 promotes ICI anti-tumor effects

**FIGURE 2:**
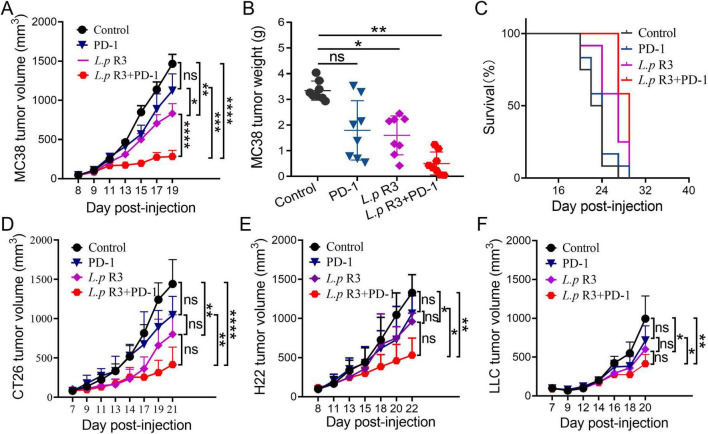
*Lactobacillus paracasei* R3 (*L.p* R3) improved checkpoint inhibitor immunotherapy. Tumor growth **(A)**, tumor weights **(B)**, and survival **(C)** of MC38-tumor-bearing mice orally administered with *L.p* R3 or phosphate-buffered saline (PBS) and treated with or without anti-PD-1 starting on day 7 (*n* = 8 mice/group). Tumor growth of CT26-tumor-bearing **(D)**, H22-tumor-bearing **(E),** or LLC-tumor-bearing **(F)** mice orally administered with *L.p* R3 or PBS and treated with or without anti-PD-1 starting on day 7 (*n* = 8 mice/group). ANOVA analysis with unpaired-t-test. (*p < 0.05, **p < 0.01, ***p < 0.001, ****p < 0.0001, ns, not significant).

To verify the improvements of *L.p* R3 to efficacy of ICI in anti-tumor treatment, MC38 colon cancer mouse model was established to assess the effects of oral administration of *L.p* R3 and combinatorial treatment of *L.p* R3 with PD-1. Oral administration of PD-1 has shown a low inhibitory effect at 22.99% (*P* = 0.0292), while oral administration of *L.p* R3 gave a higher inhibition at 60.20% (*P* < 0.0001). The combinatorial treatment of *L.p* R3 with PD-1 gave the best result, an 82.01% inhibition was obtained in the combinatorial treatment (Figure 2A). Meanwhile, mice receiving combinatorial treatment showed a significant decrease in tumor weight in the comparison with control group (Figure 2B). With regard to median survival time of MC38-tumor-bearing mice, a median survival time of 23 days was observed in control group, while the median survival time after treatment of PD-1, *L.p* R3, or PD-1 + *L.p* R3 in the MC38-tumor-bearing mice were 24, 26, and 29 days, respectively (Figure 2C).

To assess the non-specificity in the improvement of *L.p* R3 to MC38 colon cancer mouse model, CT26 colon cancer, H22 liver cancer, and LLC lung cancer mouse model were further established to assess oral administration of *L.p* R3 and combinatorial treatment of *L.p* R3 with PD-1 in other tumor treatments. In terms of the CT26 colon cancer mouse model, oral administration of PD-1 or *L.p* R3 gave inhibitory effects on tumor at a low rate (15.6% or 55.3%, respectively). Meanwhile, the best performance of anti-tumor efficacy was given by combinatorial treatment with *L.p* R3 and PD-1, which achieved 80.1% (*P* ≤ 0.0001) (Figure 2D). For the tumor in H22 liver cancer mouse model, there was 33.19% (*P* = 0.0314) or 43.04% (*P* = 0.0079) inhibition by single-drug therapy of PD-1 or *L.p* R3, meanwhile, the inhibition was 70.75% (*P* = 0. 0.00024) for combinatorial treatment of *L.p* R3 with PD-1 (Figure 2E). Likewise, the low inhibitory effects (24.8%, *P* = 0.0821) or (37.6%, *P* = 0.0093) was achieved by oral administration of PD-1 or *L.p* R3 with regard to the tumor in LLC lung cancer mouse model. Combinatorial treatment of *L.p* R3 with PD-1 gave the best performance, leading to 53.3% (*P* = 0.0005) in tumor inhibition (Figure 2F). No weight loss was observed during treatment (Supplementary Figure 1).

### 2.3 The distribution and metabolism of *L.p* R3 in mice

**FIGURE 3:**
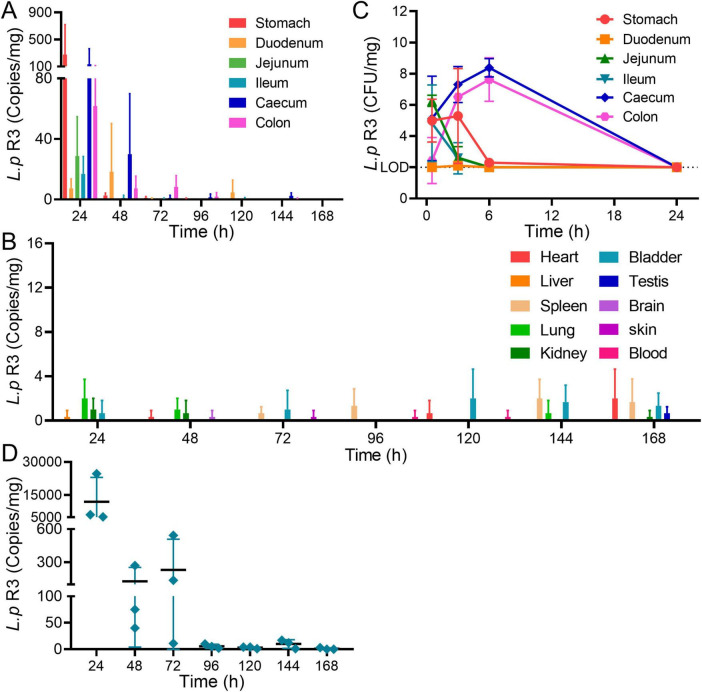
**(A)** Relative abundance of 16S rRNA copies of *Lactobacillus paracasei* R3 (*L.p* R3) in gastrointestinal organs after oral administration of *L.p* R3 in mice. **(B)** CFU of *L.p* R3 per mg gastrointestinal organs in 24 h after oral administration of *L.p* R3 to mice. **(C)** Relative abundance of 16S rRNA copies of *L.p* R3 in major organs after oral administration of *L.p* R3 in mice. **(D)** Fecal clearance of *L.p* R3.

Colonization is an important characteristic of probiotics, showing the survival in body and playing an important role in triggering immune regulation. The distribution and colonization of *L.p* R3 in mice were evaluated. Healthy C57Bl/6 male mice were treated with *L.p* R3 at a concentration of 5 × 10^9^ CFU/mouse *via* daily oral gavage over a period of 21 days. After the final oral gavage, mice were dissected to collect their tissues (blood, heart, liver, spleen, lung, kidney, brain, skin, testis, bladder, stool, stomach, duodenum, jejunum, ileum, colon and cecum) to determine the distribution of *L.p* R3 in mice by using qPCR analyses. The results showed that, after the 24 h following the last oral gavage, *L.p* R3 was mainly distributed in gastrointestinal tract (Figure 3A) while no significant distribution of *L.p* R3 was detected in the heart, liver, spleen, lung, kidney, bladder, testis, brain and skin of mice (Figure 3B and Supplementary Figure 2). A dramatic reduction in *L.p* R3 content was observed in gastrointestinal tract tissues, including stomach, duodenum, jejunum, ileum, cecum, and colon, after the last oral gavage 24 h. After 96 h, only a small amount of *L.p* R3 could be detected in cecum and colon. To determine the absorption and distribution of *L.p* R3 in gastrointestinal tract, healthy mice were treated with *L.p* R3 at a concentration of 1 × 10^10^ CFU/mouse *via* oral gavage followed by a detection on *L.p* R3 in gastrointestinal tract at 0.5, 3, 6, and 24 h post administration by using count plate method. The results showed that *L.p* R3 were mainly distributed in stomach, jejunum, ileum, and cecum after 0.5 h. After 6 h post-gavage, an increased *L.p* R3 was detected in cecum and colon, while the reduction of *L.p* R3 was observed in stomach, jejunum, and ileum. By 24 h post-gavage, a remarkable reduction of *L.p* R3 was observed in mice’s gastrointestinal tracts (Figure 3C and Supplementary Figure 3). Finally, Real-time PCR was employed to detect the metabolism of *L.p* R3 in mice feces. By 24 h post-gavage, abundant *L.p* R3 was observed in mice feces, followed by a dramatic reduction in *L.p* R3 level mice feces in the 24–48 and 48–96 h samples. After 96 h, only trace amounts of *L.p* R3 remained detectable (Figure 3D).

### 2.4 *L.p* R3 interacts with intestinal epithelial cells

**FIGURE 4:**
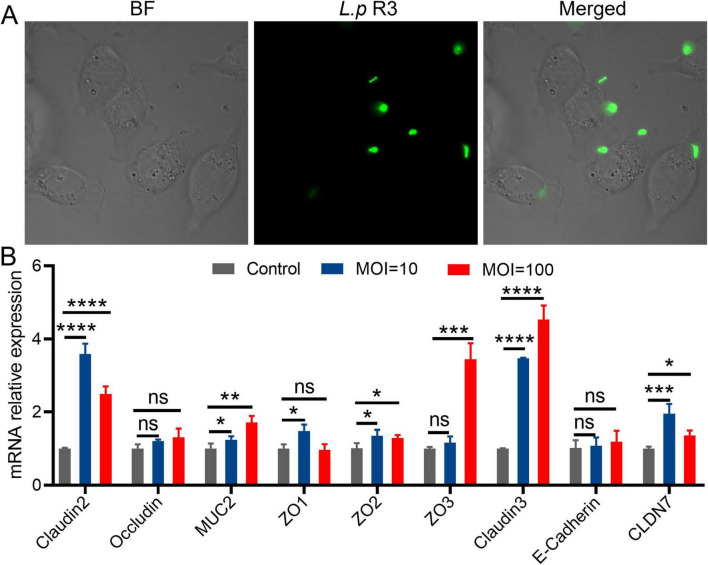
**(A)** The fluorescent images of living intestinal epithelial cells after treatment with green fluorescent protein-labeled *Lactobacillus paracasei* R3 (*L.p* R3) for 1 h. **(B)** Tight junction proteins mRNA expression in Caco-2 cells after *L.p* R3 treatment for 12 h. ANOVA analysis with unpaired-t-test. (*p < 0.05, **p < 0.01, ***p < 0.001, ****p < 0.0001, ns, not significant).

The colonization of intestinal microbiome in intestine is facilitated by its adhesion to the intestinal epithelial cells or distribution in the mucus layer. To further elucidate the mechanisms behind the colonization of *L.p* R3 in gastrointestinal tract, a experiment was undertaken to investigate the interaction between *L.p* R3 and intestinal epithelial cells. The effect of *L.p* R3 on cell viability of Caco-2 cells was assessed. The results showed that *L.p* R3 co-cultured with Caco-2 cells for 1–3 h at different multiplicity of infection (MOI, 1,000, 100, 10, 1) did not exhibit any toxicity toward the Caco-2 cells (Supplementary Figure 4). Afterward, laser confocal microscopy work was undertaken to observe the interaction between the *L.p* R3 labeled with green fluorescent protein, and Caco2 cells. The results demonstrated that the *L.p* R3, labeled with green fluorescent protein (MOI = 100), exhibited rapid adherence to the cell membrane of Caco-2 cells within 1 h of co-culturing (Figure 4A).

Given the adhesion of *L.p* R3 on the membrane of intestinal epithelial cells, whether *L.p* R3 can promote the secretion of mucoprotein in intestinal epithelial cells was verified. The mRNA expression levels of tight junction proteins in Caco-2 or goblet cells were co-cultured with *L.p* R3 (MOI = 100, MOI = 10) for 2 h and quantified by using qPCR. Significant up-regulations of mRNA expression level of Claudins (claudin2, claudin3, CLDN7), Zonula Occludens (ZO1, ZO2, ZO3) and Mucin (MUC2) in Caco-2 cells were observed followed by *L.p* R3 treatment. However, no significant differences could be detected in mRNA expression level of occludin and cadherin after *L.p* R3 treatment (Figure 4B). Similar to what was observed in enterocyte, a significant up-regulations of mRNA expression level of Claudins (claudin2, claudin3, CLDN7), Zonula Occludens (ZO1, ZO2, ZO3) and Mucin (MUC2) in goblet cell was observed followed by *L.p* R3 treatment.

### 2.5 *L.p* R3 interacts with PBMCs

**FIGURE 5:**
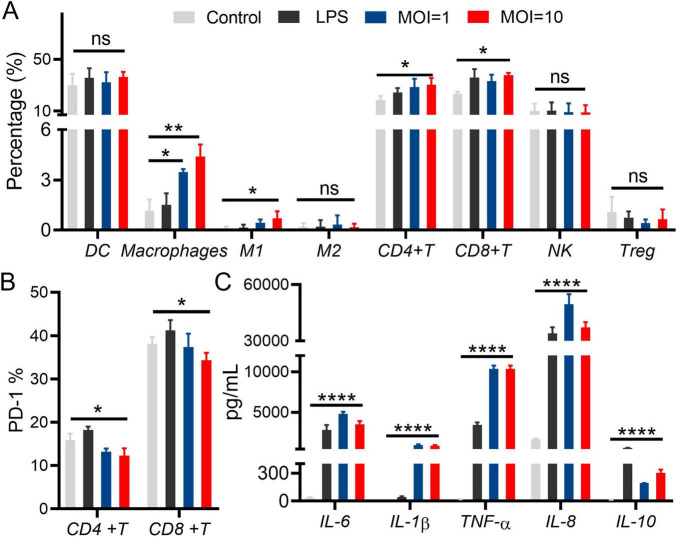
*Lactobacillus paracasei* R3 (*L.p* R3) improved checkpoint inhibitor immunotherapy. **(A)** Percentages of immune cells in peripheral monocytes after *L.p* R3 treatment were measured by flow cytometry. **(B)** PD-1 expression levels on CD4^+^T and CD8^+^T cells in peripheral monocytes after *L.p* R3 treatment. **(C)** The levels of inflammatory factors in the supernatant of peripheral monocytes treated with *L.p* R3 detected by ELISA. ANOVA analysis with unpaired-t-test. (*p < 0.05, **p < 0.01, ***p < 0.001, ****p < 0.0001, ns, not significant).

Peripheral Blood Mononuclear Cells (PBMCs) serve an important function in immune system, which differentiate along alternative lineages *in vitro* and *in vivo* depending on certain conditions, showing a preservation to host’s homeostasis. The effect of *L.p* R3 on cell viability of PBMCs was assessed. The results showed that *L.p* R3 co-cultured with PBMCs for 2 h at different multiplicity of infection (MOI, 10, 1) did not exhibit any toxicity toward the PBMCs (Supplementary Figure 5). To verify whether *L.p* R3 triggers immune system by regulating PBMCs, flow cytometer was employed to evaluate *L.p* R3 effects on PBMC differentiation. The numbers of macrophages, po-inflammatory macrophages M1, CD4^+^T cells, and CD8^+^T cells were significantly increased after 24 h of co-incubation of *L.p* R3 with PBMCs, which is similar to the differentiation after the co-incubation of Lipopolysaccharides (LPS, an Immune activator) and PBMCs (Figure 5A). The expression level of immunosuppressive molecules PD-1 on CD4^+^T cells, and CD8^+^T cells were significantly downregulated after 24 h of co-incubation of L.p R3 with PBMCs (Figure 5B). Afterward, culture supernatant of PBMCs co-incubation with *L.p* R3 was collected to further analyze the alternation of cytokines including IL-6, IL-1β, TNF- α, IL-8, and IL-10 by using ELISA. The expression levels of immune cell cytokines were all significantly increased after the co-incubation of *L.p* R3 with PBMCs (Figure 5C).

### 2.6 The effect of *L.p* R3 on serum metabolites in mice

**FIGURE 6:**
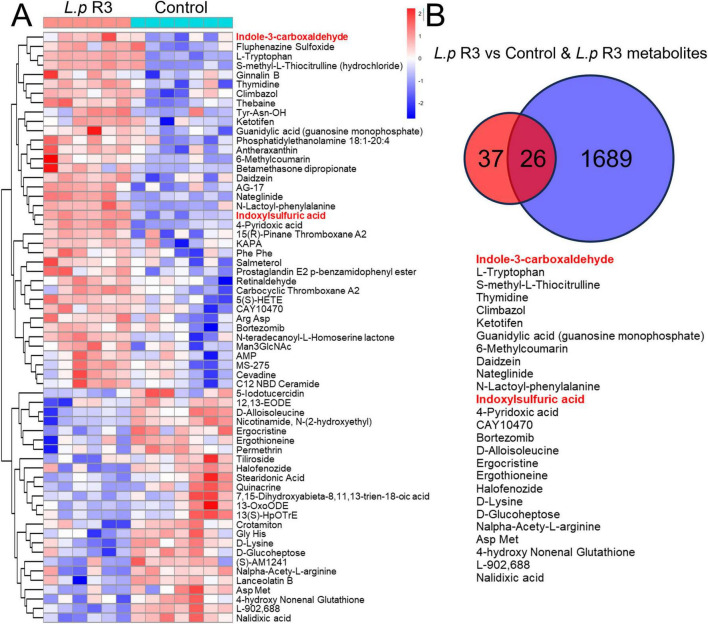
**(A)** Heat map of differential metabolites in the serum of oral *Lactobacillus paracasei* R3 (*L.p* R3) versus PBS mice. **(B)** Shared metabolites between the differential metabolites in the serum of oral *L.p* R3 versus PBS mice and *L.p* R3 metabolites.

To determine the promotion of *L.p* R3 in anti-tumor immunotherapy, an metabonomic analysis was undertaken in the serum collected from mice treated with *L.p* R3 *via* oral gavage. Compared to those mice treated with MRS, a significant up-regulation was detected in 39 kinds of serum metabolites in those treated with *L.p* R3, with indole-3-formaldehyde and tryptophan being the most highly upregulated metabolites (Figure 6A and Supplementary Figure 6). In addition, abundant indole-3-formaldehyde and tryptophan were detected in the supernatants after fermentation of *L.p* R3 in metabolites analysis, suggesting the massive production of indole-3-formaldehyde and tryptophan through tryptophan metabolism (Figure 6B). KEGG analysis revealed a high enrichment of differential metabolites related to tryptophan in several pathways, including those of the digestive system, nervous system, development and regeneration, global and overview maps, chemical structure transformation maps, amino acid metabolism, biosynthesis of other secondary metabolites, infectious disease: parasitic, Cancer: overview, and translation (Supplementary Figure 7).

## 3 Discussion

This study shows that oral treatment of *L.p* R3 significantly inhibited the development of MC38 tumor in mice, moreover, the combinatorial treatment of *L.p* R3 with PD-1 could significantly promote immune therapy and extend lifespan of MC38 tumor-bearing mice. Interestingly, this improved immune therapy showed a broad-spectrum anti-tumor activity that it not only enhanced therapy of MC38 tumor in mice, but also that of CT26 tumor, H22 tumor, and LLC tumor.

Colonization is an important characteristic of probiotic, which shows the survival of bacteria strain in the human, and it is considered to be an important factor for immune regulation (Han et al., 2021). The present study shows that *L.p* R3 could colonize colon and cecum of mice. Administration of *L.p* R3 for a continuous 7 days period results in the distribution of *L.p* R3 in colon and cecum which reached the peak in concentration after 6 h followed by a graduate decrease. Meanwhile, *L.p* R3 is still detectable by 96 h post-gavage, presenting the good colonization of *L.p* R3.

Previous studies have demonstrated that beneficial bacteria, such as Bifidobacterium and Lactobacillus, exhibit adherence to the surface of intestinal epithelial cells or distributed within the mucus layer (Deng et al., 2018; Paone and Cani, 2020). This phenomenon contributes to the establishment of a protective mucosal barrier, effectively hindering the adhesion and colonization of pathogenic bacteria. Probiotics possess the capability to stimulate the expression of mucins in intestinal epithelial cells, enhance mucus secretion, form a protective layer between mucosa and microbes, enhance and reinforce the barrier function of the intestinal mucosa. The present study shows the rapid adhesion of *L.p* R3 on membrane of intestinal epithelial cells followed by the co-incubation of *L.p* R3 with intestinal epithelial cells. Additionally, significant up-regulation of mRNA expression level of Claudins (claudin2, claudin3, CLDN7), Zonula Occludens (ZO1, ZO2, ZO3) and Mucin (MUC2) in Caco-2 cells was observed followed by *L.p* R3 treatment. This result suggests that the reason behind the colonization of *L.p* R3 in intestinal tract maybe due to the massive secretion of adhesion factors by intestinal epithelial cells triggered by rapid adhesion of *L.p* R3 on membrane of intestinal epithelial cells.

Gut microbiota serves a function in host immunity regulation while the regulation of GM has been possible to contribute to anti-tumor treatments (Khongrum et al., 2023). CD8^+^T cells were considered extremely important in anti-tumor immunity (Park et al., 2023), which have been reported to be responsible for tumor cells elimination while CD8^+^T cells function was regulated by myeloid cells and CD4^+^T cells (Ferris et al., 2020). However, it remains unclear whether the microbiome directly regulates the functionality of anti-tumor cytotoxic T cells or indirectly modulates the response of CD8^+^T cells through myeloid cells or Th1 and Th17 cells. Further investigation is required to elucidate the regulation of the anti-tumor CD8^+^T cell response by the gut microbiome. The present study suggests that *L.p* R3 can promote the differentiation of PBMCs into immune cells (macrophages, especially macrophage M1) and Cytotoxic T cells (CD4^+^T, CD8^+^T cells).

The immune inhibitory molecule PD-1 has been reported to be associated with T lymphocyte exhaustion and functional dysregulation (Bod et al., 2023). Excessive expression of PD-1 on the surface of T lymphocytes can impair their proliferation ability and killing activity (Kim et al., 2023). Our results demonstrate that *L.p* R3 effectively reduces the expression of PD-1 on both CD4^+^T cells, and CD8^+^T cells, thereby enhancing the cytotoxicity of T lymphocytes. Meanwhile, *L.p* R3 significantly upregulated the expression levels of immune cytokines including IL-6, IL-1β, TNF- α, IL-8, and IL-10. PBMCs could be the targets in anti-tumor treatment to promote host immune response by regulating the differentiation of PBMCs or enhancing the immune cell function. Further investigation will be required to investigate the potential effects of *L.p* R3 on immune cells.

Increasing attention has been given to the effects of GM on anti-tumor treatment that GM and its metabolites have the potential to exert an anti-cancer effect through modulation of immune cell activities (Fang et al., 2022; He et al., 2021). For example, short-chain fatty acids (SCFAs) produced by microorganisms regulate the response of CD8^+^T cells and improve the adaptive immunotherapy for cancer treatment. Indoles and their derivatives derived from tryptophan microbial metabolism have been reported to be therapeutic targets in diseases such as cancer and neurodegenerative diseases. Most indole derivatives, such as ILA, IAA, and I3C, are endogenous ligands of the aromatic hydrocarbon receptor (AHR), while AHR signaling pathway can regulate immune responses and help to treat neurodegenerative diseases and tumors. The results of this study show that *L.p* R3 could produce massive indole-3-formaldehyde by tryptophan metabolism. In addition, the expression level of indole-3-formaldehyde in mice serum was significantly upregulated after the oral treatment of *L.p* R3. It is inferred from these results that indole-3-formaldehyde may be the key metabolite which enhances the anti-tumor efficacy by ICIs. Whether indole-3-formaldehyde can regulate immunoreaction through AHR signal transduction pathway, warrants further investigation.

## 4 Conclusion

In summary, the present study reveals that *L.p* R3 significantly promotes ICI therapies for various tumors. The presence of *L.p* R3 was detectable in colon and cecum at 96 h after the oral treatment. The long maintenance may be due to the rapid adhesion of *L.p* R3 on the membrane of intestinal epithelial cells, which promoted the mucin secretion of intestinal epithelial cells and contributed to *L.p* R3 colonization in intestinal tract. In addition, the co-incubation of *L.p* R3 could significantly elevate the levels of macrophages and natural killer T cells and decrease the level of PD-1, which is considered to be the key factor for *L.p* R3 to promote anti-tumor immunity. Finally, an up-regulation of indole-3-formaldehyde was observed in mice serum after the oral treatment of *L.p* R3. Overall, the present study demonstrates that *L.p* R3 can enhance ICI therapy through immunomodulation, which provides potential therapeutic strategies to explore for improved ICI treatment.

### 4.1 Limitations of the study

Despite the study’s significant findings on *L.p* R3 enhancing ICI therapy, it has multiple limitations.

1. Microbiome understanding is limited. The study doesn’t fully explore *L.p* R3’s impact on the gut microbiome or its complex interplay with ICI therapy.
2. Long-term follow-up data is lacking. Short-term outcomes are presented, but long-term data is crucial to assess sustained effects, gut microbiome changes, and treatment safety/efficacy.
3. Probiotic administration is oversimplified. Focusing only on oral delivery ignores how other routes (e.g., intravenous, intertumoral) could affect the potential of *L.p* R3.
4. Mechanistic details of indole-3-formaldehyde are insufficient. The link between indole-3-formaldehyde, AHR activation, and immune responses needs clarification with functional assays.
5. Distribution and colonization data are inadequate. Existing data lack temporal kinetics, and more precise quantification of bacterial adherence is needed.

Overall, further research is required to fully understand the potential of *L.p* R3 in ICI therapy and for clinical translation.

## 5 Materials and methods

### 5.1 Cell culture

The MC38, CT26, H22, LLC, Caco-2, and HT-29 cells were purchased from American Type Culture Collection (ATCC) and cultured using the standard conditions according to ATCC instructions. MC38 and CT26 cells were cultured in RPMI 1640 medium supplemented with fetal bovine serum (10%) and penicillin-streptomycin (1%), and H22 and LLC cells were cultured in Dulbecco’s modified Eagle’s medium supplemented with fetal bovine serum (10%) and penicillin-streptomycin (1%). Caco-2 and HT-29 cells were cultured in DMEM-H medium (containing glutamine and sodium pyruvate) supplemented with fetal bovine serum (10%) and penicillin-streptomycin (1%).

### 5.2 *L.p* R3 strain and culture

*Lactobacillus paracasei* R3 was isolated from baby feces and identified by API 50CH strips (BioMerieux) and 16S rDNA sequence. *L.p* R3 strain was always activated and cultured in MRS medium in aerobic conditions at 37°C before feeding animals.

### 5.3 Tumor models

Specific pathogen free (SPF) C57BL/6 male mice (6–8 weeks-old; 19–22 g body weight) were purchased from Guangdong Medical Laboratory Animal Center, China. The mice were maintained in SPF environment under a 12/12 h photoperiod and 22 ± 2°C. Prior to the experiment, the mice were separated into five mice per cage and fed ad libitum (20% protein + 70% carbohydrates + 10% fat) and persisted for 1 week. All experiments were conducted with prior approvals from Animal ethics committee. Afterward, the cell densities of tumor cells (MC38, CT26, H22, LLC) were adjusted to 5 × 10^5^/mL and subcutaneously injected into right inguinal region of C57BL/6 mice in doses of 0.1 mL.

### 5.4 Administration of *L.p* R3 and antibiotics

After tumor inoculation, the mice were randomly divided into four groups (Control, *L.p* R3, PD-1, and *L.p* R3 + PD-1), with each group consisting of 12 mice. Those mice in the *L.p* R3 and *L.p* R3 + PD-1 groups were oral administered of *L.p* R3 solution at a concentration of 10^9^ CFU/mL, with doses of 100 μL per mouse given daily after tumor inoculation. Meanwhile, those mice in PD-1 and *L.p* R3 + PD-1 groups were treated by intraperitoneal injection with 200 μg anti-mPD-1 on the seventh, 10th, 13th, 16th and 19th day after the tumor inoculation. The tumors were macroscopic after 1 week, afterward, tumor sizes (length and width) were recorded every 2 days in the following period by using vernier caliper. The size of tumor was calculated as length × width^2^ × 0.5. In addition to tumor size, mice bodyweights were also measured every 2 days.

### 5.5 The distribution and metabolism of *L.p* R3 in mice

#### 5.5.1 The distribution of *L.p* R3 in mice *via* continuous gavage with *L.p* R3

Healthy C57BL/6 male mice were treated with continuous oral administration of 100 μL L.p R3 over 21 days at a dose of 10^9^ CFU/mL per day. The mice were euthanized by cervical dislocation at 24, 48, 72, 96, 120, 144, and 168 h post-gavage. Following the euthanasia, the mice were dissected to collect their organs including heart, liver, spleen, lung, kidney, bladder, testis, brain, skin, blood, stomach, duodenum, jejunum, ileum, cecum, and colon. Afterward, real-time PCR was employed to assess the concentration of *L.p* R3 in each tissue.

### 5.6 The distribution of *L.p* R3 in mice *via* single gavage with *L.p* R3

Healthy C57BL/6 male mice were orally treated with 100 μL *L.p* R3 at a dose of 10^10^ CFU/mL, followed by euthanasia through cervical dislocation at 0.5, 3, 6, 12, and 24 h post-gavage. The mice were then dissected to collect their organs including stomach, duodenum, jejunum, ileum, cecum, and colon. The concentration of *L.p* R3 in each tissue was assessed by using plate count method.

### 5.7 The excretion of *L.p* R3 in feces *via* single gavage with *L.p* R3

Healthy C57BL/6 male mice were orally treated with 100 μL *L.p* R3 at a dose of 10^9^ CFU/mL. Fecal samples were collected from the mice at seven periods: 24, 24–48, 48–72, 72–96, 96–120, 120–144, and 144–168 h. The concentration of *L.p* R3 in each fecal sample was determined using the plate count method.

### 5.8 Cell toxicity of *L.p* R3 strain to intestinal epithelial cells Caco-2

The log-phase Caco-2 cells were seeded in 96-well plates at a density of 5,000 cells per well. The cells were cultured until all the Caco-2 cells were fixed then washed with phosphate-buffered saline (PBS) for three times. Afterward, 0.1 mL of *L.p* R3 strains were added to the 96-well plates at various multiplicity of infection (MOI) level (10,000, 1,000, 100, 10, 1, and 0). The 96-well plates were incubated at 37°C with 5% CO_2_ in incubator for 4 h, followed by three washes with PBS. Subsequently, each well was supplemented with 100 μL PBS and 20 μL MTT (5 mg/mL in PBS), and further incubated for another 4 h. After removing the media, the plates were replenished with 150 μL DMSO. The absorbance at 490 nm was quantified using a microplate reader.

### 5.9 *L.p* R3 interacts with intestinal epithelial cells

The log-phase Caco-2 cells were seeded in a 25 mm glass bottom dish and cultured until all the cells were fixed, followed by three washes with PBS. The green fluorescent protein-labeled *L.p* R3 bacterial solution (1 mL, 10^9^ CFU/mL), was added to the dish. The dish was then placed in an incubator maintained at 37°C with 5% CO_2_ for 1 h. Afterward, the dish underwent three washes with PBS. Finally, an additional 1 mL PBS was added to the dish. The interaction between *L.p* R3 and intestinal epithelial cells (Caco-2) was observed using a fluorescence microscope with excitation at 584 nm, and emission at 607 nm.

### 5.10 *L.p* R3 promotes intestinal epithelial cell mucin gene expression

The log-phase Caco-2 cells were seeded in 6-well plates and cultured until all the cells were fixed, followed by 3 washes with PBS. The green fluorescent protein-labeled *L.p* R3 bacterial solution (1 mL, 10^9^ CFU/mL), was added to the dish. The dish was then placed in an incubator maintained at 37°C with 5% CO_2_ for 2 h. Afterward, the dish underwent three washes with PBS. The cells were then harvested for total RNA extraction in accordance with the manufacturer’s instructions. Subsequently, the concentration, purity, and integrity of the RNA were determined. The RNA was reverse transcribed into cDNA using five All-In-one RT MasterMix, according to manufacturer’s instructions. The diluted cDNA was utilized as a template for assessing gene expression levels. Primers were designed based on the human gene sequence obtained from NCBI, Supplementary Table 1. Then, the target fragments in the sample were amplified using synthesized primers through PCR amplification. RT-PCR analysis was conducted employing UltraSYBR reagents on a fluorescence quantitative gene amplification instrument, with each sample being repeated three times. GAPDH served as an internal reference gene to normalize the expression level of the target gene. The relative expression level of the target gene was determined utilizing the 2-DDCT method.

### 5.11 *L.p* R3 regulates PBMCs differentiation

The log-phase PBMCs were seeded in 6-well plates and cultured until all the cells were fixed, followed by three washes with PBS. *L.p* R3 bacterial solution (1 mL, 10^9^ CFU/mL), was added to the plates. The plate was then placed in an incubator maintained at 37°C with 5% CO_2_ for 2 h. The cells were then harvested from the sample to assess the proportion of immune cells including DCs (CD3^+^cells), macrophages (CD11b + cells), M1 (F4/80 + CD11b + CD86 + cells), M2 (F4/80 + CD11b + CD206 + cells), CD4^+^T (CD3 + CD4 + cells), CD8^+^T (CD3 + CD8 + cells), NKs (CD3 + CD15 + CD56 + cells) and Treg cells (CD3 + CD4 + CD25 + FoxP3 + cells) by using flow cytometer. Meanwhile, flow cytometer was employed to assess the expression level of PD-1 on the surfaces of CD4^+^T cells and CD8^+^T cells. The supernatant was collected from the plates to analyze the concentration of IL-6, IL-1b, TNF-a, IL-8, and IL-10 by using ELISA according to manufacturer directions.

### 5.12 Non-targeted metabolite profiling of the culture supernatant from *L.p* R3

The *L.p* R3 strain was cultured in MRS medium for 24 h until the bacterial density reached 10^9^ CFU/mL. Subsequently, the culture was centrifuged twice at 10,000 rpm for 15 min, each time to collect the supernatant. Then, 800 μL of the supernatant was transferred to a new 1.5 mL centrifuge tube. For Metabolite extraction: 100 μL of the bacterial supernatant was mixed with 10 μL of internal standard (2-chlorophenylalanine, 0.3 mg/mL). Next, 100 μL of methanol-acetonitrile (2:1, v/v) was added to the mixture and vortexed for 1 min. The resulting mixture was incubated at −20°C for 20 min and then centrifuged at 4°C 12,000 r/min for 10 min to obtain 160 μL of the supernatant for LC-MS analysis.

### 5.13 Non-targeted metabolite profiling in mouse serum

After the treatment, blood serum samples were collected separately from each mouse in both the Control group and the *L.p* R3 treatment group, with a volume of 100 μL per mouse. Subsequently, each sample was supplemented with 10 μL internal standard (L-2-chlorophenylalanine, prepared in methanol at a concentration of 0.3 mg/mL), followed by vortexing for 10 s. Then, a protein precipitation reagent (methanol-acetonitrile, 2:1, v/v) was added to each sample in a volume of 300 μL and vortexed for 1 min. The mixture was further sonicated in an ice bath for 10 min. The mixture was then allowed to stand at −20°C for 30 min, centrifuged for 15 min at 13,000 rpm and 4°C, and 200 μL of the supernatant was extracted and used for LC-MS analysis.

### 5.14 Non-targeted metabolomics data analysis and statistics

#### 5.14.1 Quality control and substance identification

The downstream data (.d) file is imported into AbfConverter and converted into an appropriate file format (.abf) for analysis. The software MS-DIAL (ver. 4.90) is utilized to conduct peak searching, peak alignment, and other necessary data processing on the (.abf) file. Compound chromatogram matching is performed based on the first- and second-level chromatograms as well as public databases MASSBANK and METLIN, in order to obtain accurate material peak identification results for the detected samples.

The low-quality peaks in the material peak (with detection rates below 50% in both the QC sample and the total profile) are eliminated. Only the peaks identified as belonging to the same substance are retained, with preference given to the peak identified with the highest accuracy rate. The remaining peaks are designated as unidentified substances and considered as part of the total detected substance peaks for initial classification and statistical analysis.

The identification of all peaks in the sample was based on the calculation of the coefficient of variation (CV, RSD) for the QC sample. Peaks with a CV greater than 30% were excluded. Stable peaks, which were identified as substances and had an area at least five times that of the blank peak, were selected to obtain metabolite identification and relative quantification results. The stable peaks in the sample were standardized using the R package MetaboAnalystR for downstream statistical analysis.

### 5.15 Statistical analysis

The standardized data is subjected to statistical tests based on grouping, as well as the calculation of the *t*-test for the mean difference (Mann-Whitney U test).

Through R functions prcomp for principal component analysis (PCA) and MetaboAnalystR package for partial least squares discriminant analysis (PLS-DA) to obtain VIP values. The statistical significance of each metabolite between the two groups was calculated based on the *t*-test, and the fold change (FC) between the two groups was calculated. The default criterion for metabolite differential screening was set as VIP > 1.0, *P* value < 0.05, and FC ≥ 2 or FC ≤ 0.5. The statistical significance of each metabolite between multiple groups was calculated based on the Kruskal-Wallis H test, and the default criterion for metabolite differential screening was set as VIP > 1.0, *P* value < 0.05. The heatmap of differential metabolites was drawn using the Pheatmap function in the R toolkit, and the metabolite data was normalized using z-score.

## Data Availability

The original contributions presented in this study are included in this article/Supplementary material, further inquiries can be directed to the corresponding authors.
