# Supplementary material for: Colonizable probiotic Lactobacillus paracasei R3 enhances ICI therapy via modulating PBMCs differentiation
Source: Front Microbiol. 2025 Jun 4;16:1547964. doi: 10.3389/fmicb.2025.1547964 (PMC12174050; doi:10.3389/fmicb.2025.1547964)
Supplement: Supplementary file 1 [file Supplementary_file_1.docx]

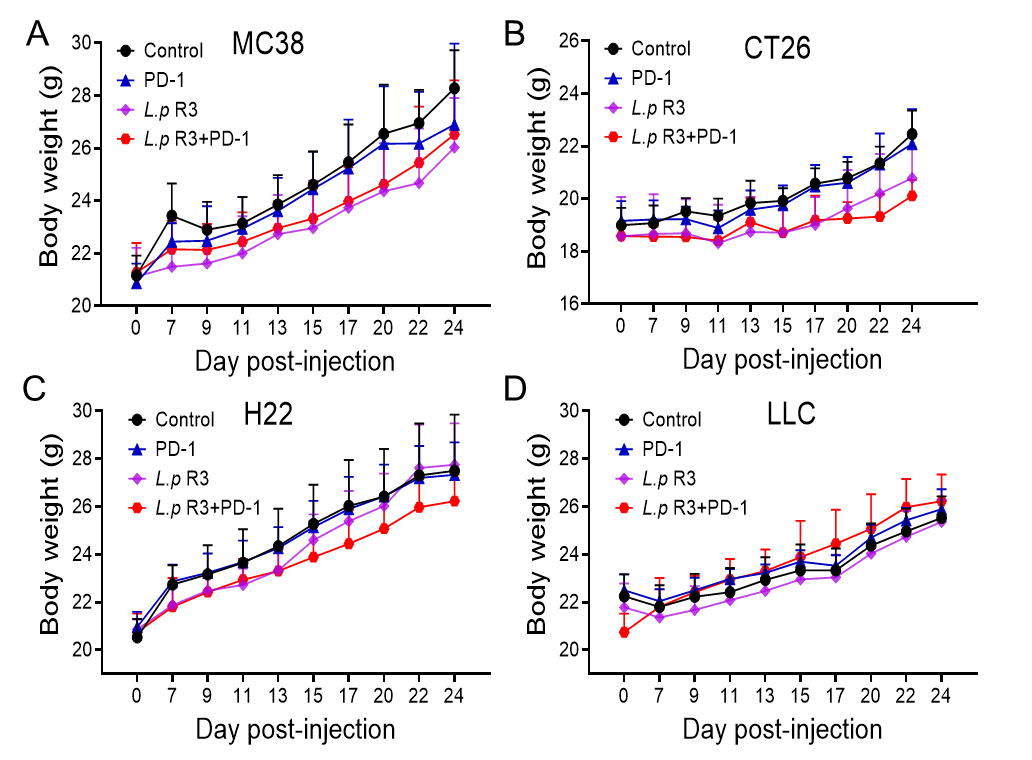


Figure S1. Effect of *L.p* R3 on the body weight of tumor-bearing mice.

Figure S2. CFU of *L.p* R3 per mg gastrointestinal organs in 24 h after oral administration of *L.p* R3 to mice.


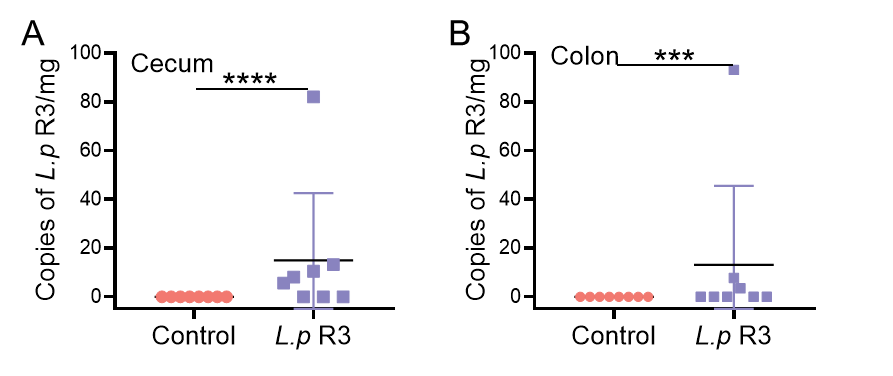


Figure S3. Relative abundance of 16S rRNA copies of *L.p* R3 in Cecum (A) and Colon (B) after oral administration of *L.p* R3 in mice

Figure S4. Viability of the Caco-2 cells was determined using MTT after stimulation with MOI 1000, 100, 10, 1 and 0 *L.p* R3 for 1 h, 2 h, and 3 h.

Figure S5. Viability of the Peripheral Blood Mononuclear cells was determined using MTT after stimulation with MOI 10, 1 and 0 *L.p* R3 for 2 h.


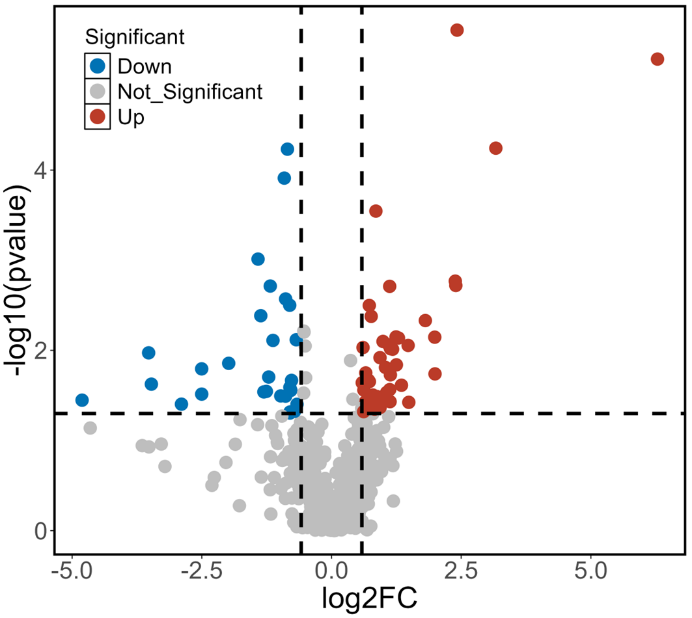


Figure S6. Heat map of differential metabolites in the serum of oral *L.p* R3 versus PBS mice.


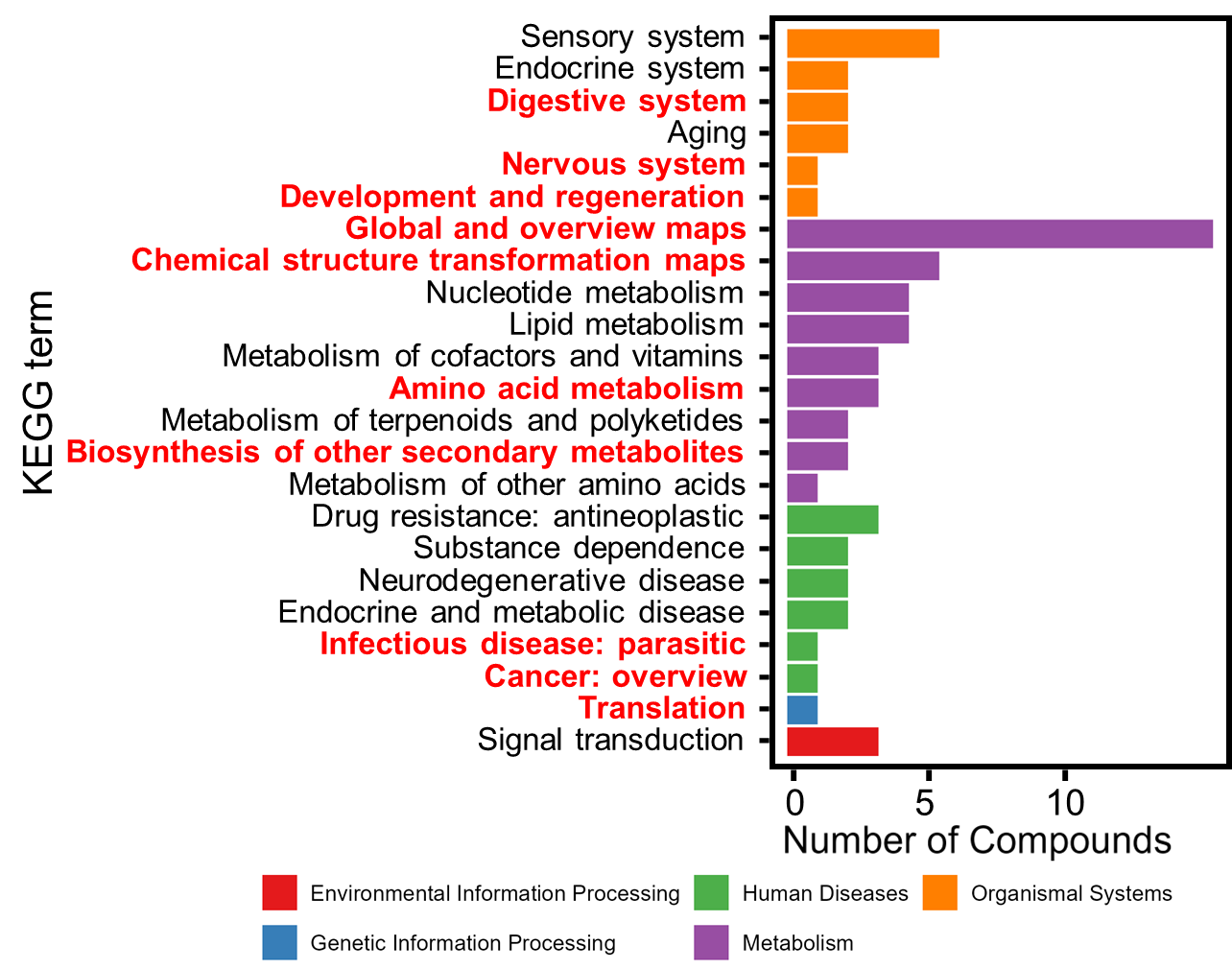


Figure S7. KEGG pathway analysis of differential metabolites.

Table S1. Primers used to quantify mRNA levels.

| Gene | FP sequence (5’-3’) | FP sequence (5’-3’) |
| --- | --- | --- |
| ZO1 | TATCCCGAGGAAATGATGAGGC | TCGTATCTGTATGTGGGCTGCT |
| ZO2 | ATGGAAGAGCTGATATGGGAACA | TGCTGAACTGCAAACGAATGAA |
| ZO3 | GCTTTGGCATTGCGATCTCTG | GATGTGGTCGCCTGTCTGTAG |
| MUC2 | ACCCGCACTATGTCACCTTC | GGACAGGACACCTTGTCGTT |
| Claudin2 | GCCTCTGGATGGAATGTGCC | GCTACCGCCACTCTGTCTTTG |
| CLDN7 | AGCTGCAAAATGTACGACTCG | GGAGACCACCATTAGGGCTC |
| Occludin | AAGAGTTGACAGTCCCATGGCATAC | ATCCACAGGCCAAGTTAATGGAAG |
| GAPDH | AACGGATTTGGTCGTATTG | ATACCAGGAAATGAGCTTGACA |
